# A novel specific grading standard study of auto-segmentation of organs at risk in thorax: subjective–objective-combined grading standard

**DOI:** 10.1186/s12938-021-00890-8

**Published:** 2021-06-03

**Authors:** Yanchen Ying, Hao Wang, Hua Chen, Jianfan Cheng, Hengle Gu, Yan Shao, Yanhua Duan, Aihui Feng, Wen Feng, Xiaolong Fu, Hong Quan, Zhiyong Xu

**Affiliations:** 1 Department of Radiation Oncology, Shanghai Chest Hospital, Shanghai Jiao Tong University, Shanghai, 200030 China; 2 Key Laboratory of Artificial Micro- and Nano-Structures of Ministry of Education and Center for Electronic Microscopy and Department of Physics, Wuhan University, Wuhan, 430070 China; 3 Institute of Modern Physics, Fudan University, Shanghai, China

**Keywords:** SOC grading standard, Auto-segmentation, Thorax, Organs at risk

## Abstract

**Background:**

To develop a novel subjective–objective-combined (SOC) grading standard for auto-segmentation for each organ at risk (OAR) in the thorax.

**Methods:**

A radiation oncologist manually delineated 13 thoracic OARs from computed tomography (CT) images of 40 patients. OAR auto-segmentation accuracy was graded by five geometric objective indexes, including the Dice similarity coefficient (DSC), the difference of the Euclidean distance between centers of mass (ΔCMD), the difference of volume (ΔV), maximum Hausdorff distance (MHD), and average Hausdorff distance (AHD). The grading results were compared with those of the corresponding geometric indexes obtained by geometric objective methods in the other two centers. OAR auto-segmentation accuracy was also graded by our subjective evaluation standard. These grading results were compared with those of DSC. Based on the subjective evaluation standard and the five geometric indexes, the correspondence between the subjective evaluation level and the geometric index range was established for each OAR.

**Results:**

For ΔCMD, ΔV, and MHD, the grading results of the geometric objective evaluation methods at our center and the other two centers were inconsistent. For DSC and AHD, the grading results of three centers were consistent. Seven OARs’ grading results in the subjective evaluation standard were inconsistent with those of DSC. Six OARs’ grading results in the subjective evaluation standard were consistent with those of DSC. Finally, we proposed a new evaluation method that combined the subjective evaluation level of those OARs with the range of corresponding DSC to determine the grading standard. If the DSC ranges between the adjacent levels did not overlap, the DSC range was used as the grading standard. Otherwise, the mean value of DSC was used as the grading standard.

**Conclusions:**

A novel OAR-specific SOC grading standard in thorax was developed. The SOC grading standard provides a possible alternative for evaluation of the auto-segmentation accuracy for thoracic OARs.

## Background

Accurate delineation of organs at risk (OARs) is an essential step in ensuring radiotherapy dosimetry accuracy. Over recent years, the auto-segmentation of OARs has gained increasing importance. Compared to cumbersome slice-by-slice manual delineation, auto-segmentation not only saves time to radiation oncologists [1] but also reduces inter- and intra-observer variations [2, 3]. A number of commercial auto-segmentation software have been developed and gradually used in clinical, such as MIM Maestro (MIMVista Corp, Cleveland, US-OH), SPICE (Philips, Madison, WI), and ABAS (CMS-Elekta, Stockholm, Sweden) [4]. However, some studies suggested that the contours generated by auto-segmentation should still be carefully reviewed by radiation oncologists [3, 5–8].

At present, the evaluation methods for the auto-segmentation accuracy for thoracic OARs have not yet been standardized. There are three major methods: the geometric objective evaluation method, which includes grading the performance of OAR auto-segmentation by geometric indexes. For instance, Velker et al. [9] graded the auto-segmentation accuracy into three levels according to Dice similarity coefficient (DSC) as: good (0.8 ≤ DSC ≤ 1), medium (0.6 ≤ DSC < 0.8), and poor (0 ≤ DSC < 0.6). Notably, Ciardo et al. [5] used three indexes of DSC, the difference of the Euclidean distance between centers of mass (ΔCMD) and average Hausdorff distance (AHD) to grade segmentation accuracy into three levels. This method is quantitative and universal, but it has no support from the subjective evaluation. The second method is the subjective evaluation method [6, 10], which grades segmentation accuracy according to the degree of modification required for the auto-segmentation contours that are judged by radiation oncologists’ clinical experience and subjective will. This method isn’t quantitative; thus, it cannot be popularized to other radiotherapy centers for grading evaluation of the auto-segmentation accuracy. The third method is the subjective and objective combined evaluation method [7]. Recent research in this area obtained the median values of the geometric indexes corresponding to the level of subjective evaluation levels based on DSC and maximum Hausdorff distance (MHD); however, they did not provide the ranges of geometric indexes; thus, this approach cannot be used as a general evaluation standard.

The above three evaluation methods have their own disadvantages. Each one has a different evaluation base, so that it is difficult to horizontally compare the auto-segmentation accuracy between different software for different OARs. Hence, it is extremely urgent to develop a uniform evaluation standard for the accuracy of auto-segmentation software. In addition, a correlation between OAR volume and geometric indexes such as DSC has been previously reported [11]. A specific grading standard for auto-segmentation accuracy is developed for each OAR, thus making research results more accurate.

The auto-segmentation algorithm has been extensively studied for regions such as the head and neck [11–13], and abdomen [14]. Considering thorax, there are also many studies reporting auto-segmentation for common OARs such as heart, lung, spinal cord, trachea, and esophagus [15–18]. Nevertheless, none of these studies have been undertaken for all the thoracic OARs listed in the Radiation Therapy Oncology Group (RTOG) delineation guidelines [19], including great vessels, chest wall, and skin. In order to make the content more comprehensive, we tried to study all the thoracic OARs listed in RTOG guidelines.

The aim of this study was to establish an OAR-specific subjective–objective-combined (SOC) grading standard in thorax for evaluating the accuracy of all commercial and self-developed auto-segmentation software. Thirteen thoracic OARs were auto-segmented, and five geometric indexes of DSC, ΔCMD, the difference of volume (ΔV), MHD, and AHD were calculated in our work. The novel OAR-specific SOC grading standard was developed by combining the subjective evaluation standard proposed by us and the geometric objective indexes. We clarified the correspondences between the subjective evaluation levels and the ranges of DSC for thoracic OARs. Consequently, the SOC grading standard should have great potential for applications in the accuracy evaluation of auto-segmentation software based on traditional algorithms and deep learning algorithms.

## Results

### Grading results by geometric indexes

Table 1 shows the results of the five geometric indexes between manual and auto-segmentation contours for 13 OARs in the thorax. Table 2 shows the grading results of 13 OARs in the thorax by the geometric objective evaluation method of our center and the other two centers. According to the DSC, we graded the right lung (R Lung), left lung (L Lung), skin, heart, and spinal cord (SC) as Level 3 (mean DSC: 0.88–0.96), which required some manual modification after auto-segmentation; the aorta (AOR), chest wall (CW), trachea, and pulmonary artery (PA) were Level 2 (mean DSC: 0.73–0.79), which required many manual modifications after auto-segmentation; the superior vena cava (SVC), esophagus (ESO), inferior vena cava (IVC), and pulmonary vein (PV) were Level 1 (mean DSC: 0.53–0.62), for which the use of auto-segmentation was not recommended. Compared with Velker et al. [9] and Ciardo et al. [5], the grading results of all the OARs were consistent except for the SVC. Moreover, the grading results of the AHD in our center were basically identical with those of Ciardo et al. [5], except for the L Lung.

**Table 1:** Five geometric indexes between manual contours and auto-segmentation contours for 13 organs at risk in thorax (mean ± SD)

Structure	DSC	ΔCMD (cm)	ΔV (%)	MHD (cm)	AHD (cm)
R Lung	0.96 ± 0.02	0.13 ± 0.07	7 ± 5	2.18 ± 0.72	0.11 ± 0.05
L Lung	0.94 ± 0.03	0.28 ± 0.28	10 ± 8	3.45 ± 2.19	0.17 ± 0.12
Skin	0.93 ± 0.06	2.06 ± 2.38	11 ± 10	8.85 ± 4.21	0.58 ± 0.54
Heart	0.90 ± 0.07	0.40 ± 0.40	7 ± 10	1.85 ± 1.11	0.24 ± 0.17
SC	0.88 ± 0.04	1.34 ± 2.01	9 ± 7	2.74 ± 4.19	0.13 ± 0.21
AOR	0.79 ± 0.10	0.92 ± 0.48	24 ± 20	2.72 ± 1.15	0.28 ± 0.16
CW	0.77 ± 0.05	1.29 ± 0.66	39 ± 26	6.51 ± 1.97	0.46 ± 0.19
Trachea	0.75 ± 0.09	0.53 ± 0.31	34 ± 51	4.27 ± 4.65	0.25 ± 0.25
PA	0.73 ± 0.09	0.71 ± 0.44	16 ± 10	2.12 ± 1.14	0.28 ± 0.17
SVC	0.62 ± 0.09	1.17 ± 0.52	29 ± 17	1.87 ± 0.79	0.33 ± 0.13
ESO	0.57 ± 0.11	0.89 ± 0.60	33 ± 33	2.10 ± 0.74	0.29 ± 0.13
IVC	0.56 ± 0.16	0.91 ± 0.62	30 ± 25	2.17 ± 1.07	0.43 ± 0.24
PV	0.53 ± 0.14	1.00 ± 0.49	35 ± 24	2.65 ± 0.90	0.44 ± 0.11

**Table 2:** Grading results of 13 organs at risk in thorax by five geometric indexes

Structure	DSC			ΔCMD		ΔV	MHD	AHD	
	Our center	Velker et al. [9]	Ciardo et al. [5]	Our center	Ciardo et al. [5]	Our center	Our center	Our center	Ciardo et al. [5]
R Lung	3	3	3	3	3	3	2	3	3
L Lung	3	3	3	3	2	3	1	3	2
Skin	3	3	3	1	1	2	1	1	1
Heart	3	3	3	3	2	3	2	2	2
SC	3	3	3	1	1	3	1	3	3
AOR	2	2	2	2	1	1	1	2	2
CW	2	2	2	1	1	1	1	1	1
Trachea	2	2	2	2	1	1	1	2	2
PA	2	2	2	2	1	2	2	2	2
SVC	1	2	2	1	1	1	2	2	2
ESO	1	1	1	2	1	1	2	2	2
IVC	1	1	1	2	1	1	2	1	1
PV	1	1	1	2	1	1	1	1	1

In addition, the grading results obtained by the ΔCMD were quite different from those of Ciardo et al. [5]. The levels of L Lung, heart, AOR, trachea, PA, ESO, IVC, and PV were higher than those of Ciardo et al. [5]. The other two centers have not studied the grading results of the ΔV and MHD, so the results of our two indexes could not be compared with other centers.

Based these results, both the DSC and AHD are suitable to be used as the main geometric indexes. Combined with the results of the third part below, we recommend the DSC as the only geometric objective index for auto-segmentation accuracy evaluation.

### Grading results by subjective evaluation standard

The grading results of 13 OARs in the thorax by the subjective evaluation standard are listed in Table 3. Level 3 OARs were the R Lung, L Lung and spinal cord (the range of average percentage to be modified: 6–10%); Level 2 OARs were the skin and heart (the range of average percentage range to be modified: 14–17%); the other eight OARs were Level 1 (the range of average percentage range to be modified: 31–75%).

**Table 3:** Grading results of 13 organs at risk in thorax by the subjective evaluation standard

Structure	Average standard slice number	Average slice number to be modified	Average percentage to be modified (%)	Subjective score
R Lung	71	5	6	3
L Lung	71	7	10	3
Skin	125	19	14	2
Heart	31	6	17	2
SC	124	11	8	3
AOR	62	27	41	1
CW	71	29	42	1
Trachea	47	15	31	1
PA	12	7	58	1
SVC	22	13	57	1
ESO	72	54	74	1
IVC	13	10	75	1
PV	9	7	–	1

The above grading results were not wholly consistent with the geometric objective evaluation results. For the skin, heart, AOR, CW, trachea, and PA in all the OARs (46.2%), the grading results of subjective evaluation standard were one level lower than those of the DSC by our center, which suggests that the geometric index cannot fully evaluate auto-segmentation accuracy.

### The SOC grading standard

The SOC grading standard of 13 OARs in the thorax is shown in Table 4. There were two cases, depending on whether the level distribution of the OAR is single. The first type of OARs was the R Lung, CW, PA, SVC, ESO, IVC, and PV. Since all cases for each of the OARs were the same level, only one range of the geometric index corresponding to the level could be determined. For example, the DSC, ΔCMD, ΔV, MHD and AHD for the R Lung of Level 3 were 0.93–1, 0–0.26 cm, 0–14%, 0–4.27 cm, 0–0.19 cm, respectively.

**Table 4:** The SOC grading standard of 13 organs at risk in thorax

Structure	DSC			ΔCMD (cm)			ΔV (%)			MHD (cm)			AHD (cm)		
	1	2	3	1	2	3	1	2	3	1	2	3	1	2	3
R Lung	–	–	**0.93–1**	–	–	**0–0.26**	–	–	**0–14**	–	–	**0–4.27**	–	–	**0–0.19**
L Lung	**–**	**0.88-0.95**	**0.95–1**	**–**	0.45	0.10	**–**	16	4	–	4.97	1.93	–	0.26	0.08
Skin	**0–0.87**	0.96	0.97	** ≥ 4.03**	1.11	0.48	** ≥ 22**	7	6	13.75	7.56	7.93	** ≥ 0.91**	0.34	0.29
Heart	0.84	0.92	0.94	0.75	0.28	0.14	14	5	2	2.77	1.46	1.43	0.39	0.19	0.14
SC	**0–0.84**	0.87	**0.89–1**	4.38	1.91	**0–0.76**	19	12	6	6.58	3.67	1.51	0.40	0.14	0.05
AOR	**0–0.87**	**0.88-0.89**	**0.94–1**	** ≤ 0.20**	0.57	0.20	29	8	6	3.01	1.56	1.50	** ≥ 0.14**	**0.10-0.13**	** ≤ 0.07**
CW	**0–0.85**	–	–	** ≥ 0.28**	–	–	** ≥ 2**	–	–	** ≥ 3.60**	–	–	** ≥ 0.25**	–	–
Trachea	0.72	0.81	–	0.56	0.44	–	43	8	–	4.87	2.48	–	0.29	0.12	–
PA	**0–0.89**	–	–	** ≥ 0.21**	–	–	** ≥ 1**	–	–	** ≥ 1.11**	–	–	** ≥ 0.10**	–	–
SVC	**0–0.73**	–	–	** ≥ 0.35**	–	–	** ≥ 1**	–	–	** ≥ 0.95**	–	–	** ≥ 0.17**	–	–
ESO	**0–0.72**	–	–	** ≥ 0.26**	–	–	** ≥ 1**	–	–	** ≥ 0.95**	–	–	** ≥ 0.15**	–	–
IVC	**0–0.81**	–	–	** ≥ 0.22**	–	–	** ≥ 1**	–	–	** ≥ 0.89**	–	–	** ≥ 0.12**	–	–
PV	**0–0.74**	–	–	** ≥ 0.38**	–	–	** ≥ 2**	–	–	** ≥ 1.18**	–	–	** ≥ 0.20**	–	–

**Fig.1:**
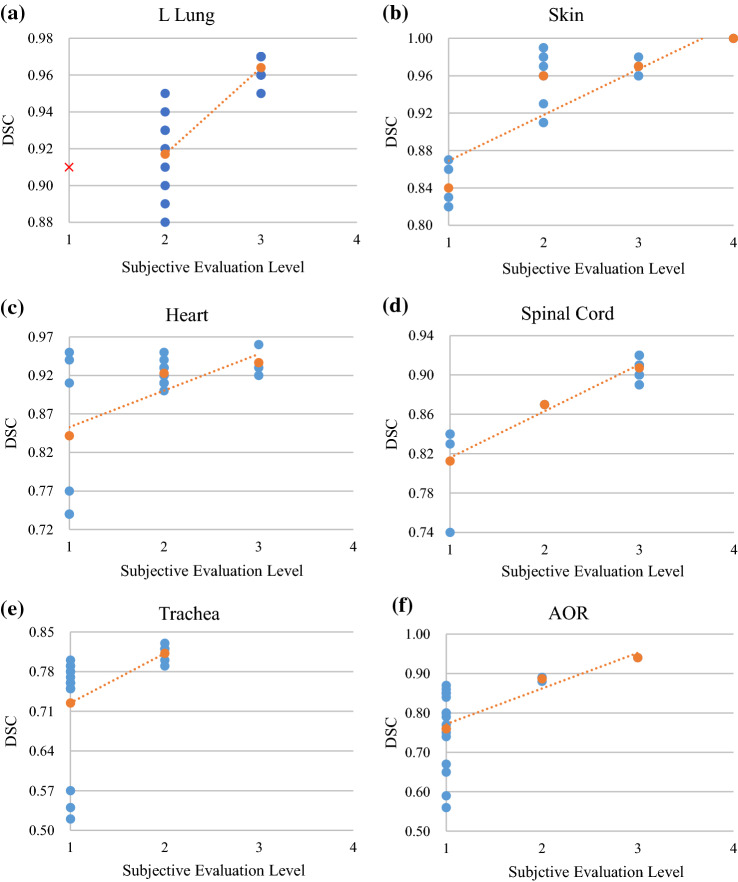
Correspondences between the subjective evaluation levels and the Dice similarity coefficients (DSC) of six organs at risk in the thorax. Blue represents the DSC value of each patient, and orange represents the mean DSC corresponding to each subjective evaluation level. The dotted line represents the linear trend line of the mean DSC. Red in (a) represents an outlier. It was observed that the auto-segmentation contour not only contained the Lung accurately, but also contained the empty stomach, which could be quickly deleted. So, this auto-segmentation contour still saved a lot of time compared to manual contour, which could be graded as Level 2

The second type of OARs was the L Lung, skin, heart, spinal cord, AOR, and trachea, and their level distribution wasn’t single. Figure 1 shows the correspondences between the subjective evaluation levels and the DSCs of the six OARs. For the L Lung, spinal cord, and AOR, the DSC ranges corresponding to the partial levels for each OAR did not overlap, which were used as the grading standard; for the other OARs or levels, the DSC ranges corresponding to the different levels overlapped. DSC's mean values still increased with the increase of the subjective evaluation levels, so the mean values were selected as the grading standard. In addition, the correspondences between the other four geometric indexes and the levels of the six OARs were less obvious than those of the DSC. So, the four indexes were not suitable as geometric objective evaluation indexes for the OAR auto-segmentation accuracy.

Although the first type of OARs can use all the five indexes to evaluate segmentation accuracy, the second type of OARs is not suitable for evaluation using all indexes. Combined with the results from the first part, we finally chose the DSC as a component of the evaluation indexes in the SOC grading standard.

## Discussion

Thanks to computer technology advancements, the auto-segmentation software based on traditional algorithms and deep learning for OARs has undergone continuous development [22, 23]. More time is needed to evaluate the segmentation accuracy, although the software can perform auto-segmentation. At present, some studies have reported on the evaluation methods for the accuracy of auto-segmentation software [5–7, 9, 10]; however, their evaluation bases are different. Therefore, it is especially important to develop a uniform evaluation standard for the software's auto-segmentation accuracy.

In this paper, 13 thoracic OARs were auto-segmented by MIM software. Five geometric indexes of DSC, ΔCMD, ΔV, MHD, AHD, and the subjective evaluation level were used as the evaluation indexes of auto-segmentation accuracy. This is the first study that proposed an easy-to-operate subjective evaluation standard to the best of our knowledge. In order to improve the consistency of evaluation, we adopted the subjective–objective-combined evaluation method. In this way, the geometric index range corresponding to the subjective evaluation level of each thoracic OAR was found, which was a new OAR-specific SOC grading standard. The SOC grading standard can be used to assess the auto-segmentation accuracy by the value of the geometric index. The standard has more clinical universality due to the diversity of thoracic OARs. For other clinical treatment sites, the standard can provide theoretical guidance and research ideas.

The geometric objective evaluation method of auto-segmentation accuracy assesses the difference between auto-segmentation and manual contours according to the geometric index. Five geometric indexes were used in this paper (Table 2). Velker et al. [9] used the DSC, and the grading results obtained by the DSC were consistent with ours. Ciardo et al. [5] used the DSC, ΔCMD, and AHD. Although their grading results obtained by the DSC and AHD were consistent with ours, their grading results of the ΔCMD were quite different from ours. Dolz et al. [24] calculated the ΔV and MHD, but they did not grade accuracy according to the two indexes. The papers mentioned above used atlas-based auto-segmentation method which were the same as ours. On the one hand, there are not enough studies that graded segmentation accuracy by the ΔV and MHD, thus making it challenging to compare the grading results. On the other hand, the two indexes' grading results were quite different from those of the DSC. Therefore, the two indexes may not be suitable as evaluation indexes, thus suggesting the use of DSC and AHD as the main geometric evaluation indexes. As shown in Table 4, the correspondence between the AHD and the subjective evaluation level is not strong. Hence, the DSC was selected as a component of the evaluation index in the SOC grading standard.

Lustberg et al. [7] reported significant geometric difference between the manual and user-adjusted contour of the esophagus, while both were accepted with local clinical guidelines by the radiation oncologists. Similarly, our results (Tables 2, 3) also showed that the geometric indexes’ results were not completely identical to those of subjective evaluation standards. The main reason is that some slices with geometric differences may be subjectively considered do not need to be modified. Therefore, it is likely that the evaluation standard of segmentation accuracy based on geometric indexes alone is not accurate. Morris et al. [6] and Zhu et al. [10] adopted the reliable subjective evaluation method. The radiation oncologist evaluated the auto-segmentation accuracy according to the coincidence degree between the auto-segmentation contours and the anatomical structure of OAR. However, their results cannot be used directly for other centers because of the lack of specific operating procedures.

Based on the above studies, we proposed an easy-to-operate subjective evaluation standard for three different length types of OAR (Table 5). Using this standard, the segmentation accuracy can be directly graded by the number or percentage of CT slice to be modified. The slice numbers of thoracic OARs except for PV (average slices: 10) in this study are more than ten slices. The evaluation standard for the < 3 slices may apply to small-volume OARs in the head and neck.

**Table 5:** Evaluation methods of the auto-segmentation accuracy by geometric indexes in 4 studies

	DSC				ΔCMD(cm)				ΔV(%)				MHD(cm)				AHD(cm)			
Level	1	2	3	4	1	2	3	4	1	2	3	4	1	2	3	4	1	2	3	4
Our center (mean value)	0–0.7	0.7–0.8	0.8–1.0	1	> 1.0	0.5–1.0	0–0.5	0	> 20	10–20	0–10	0	> 2.2	1.0–2.2	0–1.0	0	> 0.4	0.2–0.4	0–0.2	0
Velker et al. [9] (mean value)	0–0.6	0.6–0.8	0.8–1.0	–	–	–	–	–	–	–	–	–	–	–	–	–	–	–	–	–
Ciardo et al. [5] (median value)	0–0.6	0.6–0.8	0.8–1.0	–	> 0.5	0.2–0.5	0–0.2	–	–	–	–	–	–	–	–	–	> 0.40	0.15–0.40	0–0.15	–
Lustberg et al. [7] (median value)	0.57	0.89	0.91	0.98	–	–	–	–	–	–	–	–	2.6	1.7	1.1	0.4	–	–	–	–

Compared with the geometric objective evaluation methods, the subjective evaluation methods by experienced radiotherapists are more reliable. However, the grading results of different radiation oncologists may be different. Especially the grading results of the junior radiation oncologists are more accurate than the senior radiation oncologists. We selected an experienced oncologist as the expert to evaluate the all contours. In this way, inter-observer variations will be reduced, and the consistency of grading results will be improved. The auto-segmentation software for different OARs has different performance. The level distribution for the first type of OARs was single, which included the R Lung, CW, PA, SVC, ESO, IVC, and PV. For these OARs, we can only determine the DSC range corresponding to only one level (Table 4). Further investigation is needed to improve their grading standard, except for the R Lung. With the continuous improvement of algorithms and imaging methods, the auto-segmentation accuracy of these poor-performance OARs may be better. The DSC range, which corresponds to higher levels, will be supplemented in the future. In addition, the level distribution of the second OARs was not single, which included the L Lung, skin, heart, spinal cord, AOR, and trachea. The correspondences between their partial, subjective evaluation levels and the DSC ranges were clear and were used for each thoracic OAR to evaluate segmentation accuracy. For the other OARs, the DSC ranges of adjacent levels were overlapped, so the mean values were selected as the grading standard.

It was found that more ranges of the other four geometric indexes except DSC between adjacent levels were not continuous. A possible explanation for this might be insufficient sample. Due to limitations of computed tomography (CT) contrast and resolution, it is even difficult for the radiation oncologist to distinguish where the contours of some OARs should be, such as brachial plexus. It was graded directly as Level 1, which was not recommended for auto-segmentation.

The SOC grading standard proposed in this paper generated preliminary results. The research method is applicable for accuracy evaluation of OARs or tumors auto-segmentation in the thorax. Other radiation centers can directly use the SOC standard for auto-segmentation evaluation or obtain their standards by using our method. These grading standards are applicable to the traditional algorithm and auto-segmentation based on deep learning, which is the future developmental direction. On the one hand, these grading standards have great potential to assist radiation oncologists in evaluating the accuracy of OAR auto-segmentation, guiding the clinical use of the auto-segmentation software, and ensuring the accuracy of treatment planning evaluation. On the other hand, these standards might further the resolution of the lack of standardized evaluation methods of auto-segmentation accuracy. It will make the accuracy comparison between different auto-segmentation softwares more meaningful, thereby improving meta-analysis reliability.

## Conclusions

In this work, a novel OAR-specific SOC grading standard in thorax was developed. Compared with the current geometric objective evaluation method and subjective evaluation method, the SOC grading standard represents some improvement in the accuracy evaluation of auto-segmentation software. So, the SOC grading standard provides a possible alternative to evaluating auto-segmentation accuracy based on deep learning and traditional algorithms for thoracic OARs.

## Methods

### Patient selection and generation of manual contours

A total of 40 patients with thoracic malignant tumors treated in our center between November and December 2018, including patients with lung, esophageal, and thymic tumors, were retrospectively selected. The dataset included 12 females and 28 males. The median age of the dataset was 61 years (range 16–78 years). CT scans of each patient were obtained by a Siemens Somatom Definition AS CT Scanner System (Siemens Healthcare, Erlangen, Germany). The slice thickness of CT scans was 3 mm. The images were transferred to Pinnacle^3^ treatment planning system (TPS) v9.10 (Philips Healthy, Fitchburg, WI, USA).

Following the RTOG guidelines, a trained radiation oncology resident in our center manually delineated 13 thoracic OARs, including L Lung, right lung, spinal cord, heart, esophagus, chest wall, aorta, pulmonary artery, pulmonary vein, superior vena cava, inferior vena cava, skin and trachea of forty patients on the Pinnacle TPS.

Forty patients were randomly divided into two groups, the training dataset (20) and the test dataset (20). The patients in the two datasets did not intersect. The manual contours of the training dataset were used for training the auto-segmentation algorithm. The manual and auto-segmentation contours of the test dataset were used for evaluating the auto-segmentation accuracy of the algorithm.

### Generation of auto-segmentation contours

Firstly, CT images and structures of the training dataset were transferred to MIM 6.8.7. By using these, we created an atlas library of thoracic OARs for training the auto-segmentation capability of MIM. After the training was completed, the CT images of the test dataset were transferred to MIM. Their auto-segmentation contours were obtained with the simultaneous truth and performance level estimation (STAPLE) algorithm. The manual contours were used as the gold standard to evaluate the auto-segmentation accuracy of thoracic OARs.

### Main evaluation methods by geometric indexes

At present, there are three major methods for evaluating the auto-segmentation accuracy of thoracic OARs: (1) geometric objective evaluation method, (2) subjective evaluation method, and (3) subjective and objective combined evaluation method. As shown in Table 5, the first and third methods involved geometric indexes.

We evaluated the auto-segmentation accuracy of thoracic OARs by five indexes of DSC, ΔCMD, ΔV, MHD, and AHD before, as shown in Table 5. The DSC is defined as ${\text{DSC = }}\frac{{{2}\left| {V_{{{\text{manual}}\,\,}} \cap } \right.\left. {V_{{{\text{atlas}}}} } \right|}}{{\left| {V_{{{\text{manual}}\,\,}} \cap } \right.\left. {V_{{{\text{atlas}}}} } \right|}},$ where $V_{{{\text{manual}}}}$ means the volume of manual contour, and $V_{{{\text{atlas}}}}$ means the volume of atlas contour [20]. The range of DSC is 0–1; where 1 represents the two perfectly coincident contours. The MHD and AHD are the maximum and average distance between two point sets of the two contours, respectively [21]. The smaller the MHD and AHD, the smaller the difference between the two contours.

### Main evaluation methods by subjective scoring

The subjective evaluation method and the subjective and objective combined evaluation method both involved subjective scoring of the auto-segmentation contours by the radiation oncologists listed in Table 6.

**Table 6:** Evaluation methods of the auto-segmentation accuracy by subjective scoring in 3 studies

Level	1	2	3	4	5
Morris et al. [6]	Clinically unacceptable	Major modifications required	Moderate modifications required	Minor modifications required	Clinically acceptable
Zhu et al. [10]	Useful as autocontoured	Useful with minor edits	Not useful	–	–
Lustbrg et al. [7]	No result is useful basis for further editing, no time saving	Some results are useful for further editing, little time saving	Many results are useful for further editing, a moderate time saving	Most results are useful for further editing, a significant time saving	–

We developed an easy-to-operate subjective evaluation standard (Table 7), which divided the auto-segmentation accuracy into four following levels: poor, moderate, good, and great consistency between auto-segmentation and manual contours. Considering that different OARs have different lengths, we discussed three cases according to the CT slices of OAR: > 10 slices, 3–10 slices, and < 3 slices.

**Table 7:** Easy-to-operate subjective evaluation standard of the auto-segmentation accuracy

Level	Auto-segmentation performance	> 10 slices (percentage to be modified)	3–10 slices (slice number to be modified)	< 3 slices (slice number to be modified)
1	Auto-segmentation is not recommended	20%–100%	> 3	3
2	Many manual modifications are required after auto-segmentation	10%–20%	2–3	2
3	Some manual modifications are required after auto-segmentation	0–10%	1	1
4	Auto-segmentation can completely replace manual delineation	0	0	0

### The development of SOC grading standard

Based on the above subjective evaluation standard, a new SOC grading standard was proposed to overcome the shortcomings of the lack of clinician review of the geometric objective evaluation method and inter-observer variations of the subjective evaluation method. The SOC grading standard was determined by combining the subjective evaluation level of each OAR with the five geometric index ranges corresponding to the level.

The procedure of developing the SOC grading standard was as follows: first, the five geometric index values between the auto-segmentation contours and the manual contours in the test dataset were calculated. Next, an expert radiation oncologist carefully reviewed all the auto-segmentation contours slice by slice and recorded the slice number required modification. Then, all the auto-segmentation contours were graded according to the above subjective evaluation standard. Finally, the range or mean value of the geometric index corresponding to each subjective evaluation level of each OAR was observed, which represented the grading standard of auto-segmentation accuracy.

## Data Availability

The datasets used and/or analyzed during the current study are available from the corresponding author on reasonable request.

## Abbreviations

OAR: Organ at risk
DSC: Dice similarity coefficient
ΔCMD: The difference of the Euclidean distance between centers of mass
AHD: Average Hausdorff distance
MHD: Maximum Hausdorff distance
RTOG: Radiation therapy oncology group
SOC: Subjective–objective-combined
ΔV: The difference of volume
R Lung: Right lung
L Lung: Left lung
SC: Spinal cord
AOR: Aorta
CW: Chest wall
PA: Pulmonary artery
SVC: Superior vena cava
ESO: Esophagus
IVC: Inferior vena cava
PV: Pulmonary vein
CT: Computed tomography
TPS: Treatment planning system
STAPLE: Simultaneous truth and performance level estimation
